# Cooperative Localization Using Distance Measurements for Mobile Nodes

**DOI:** 10.3390/s21041507

**Published:** 2021-02-22

**Authors:** Wenchao Li, Beth Jelfs, Allison Kealy, Xuezhi Wang, Bill Moran

**Affiliations:** 1 School of Science, RMIT University, Melbourne, VIC 3000, Australia; beth.jelfs@rmit.edu.au (B.J.); allison.kealy@rmit.edu.au (A.K.); xuezhi.wang@rmit.edu.au (X.W.); 2 Department of Electrical and Electronic Engineering, University of Melbourne, Melbourne, VIC 3010, Australia; wmoran@unimelb.edu.au

**Keywords:** localization, sensor network, multidimensional scaling, position ambiguity

## Abstract

This paper considers the two-dimensional (2D) anchorless localization problem for sensor networks in global positioning system (GPS)-denied environments. We present an efficient method, based on the multidimensional scaling (MDS) algorithm, in order to estimate the positions of the nodes in the network using measurements of the inter-node distances. The proposed method takes advantage of the mobility of the nodes to address the location ambiguity problem, i.e., rotation and flip ambiguity, which arises in the anchorless MDS algorithm. Knowledge of the displacement of the moving node is used to produce an analytical solution for the noise-free case. Subsequently, a least squares estimator is presented for the noisy scenario and the associated closed-form solution derived. The simulations show that the proposed algorithm accurately and efficiently estimates the locations of nodes, outperforming alternative methods.

## 1. Introduction

Because of the increased availability of low-cost low-power sensors, smart sensors, and multi-functional sensors, wireless sensor networks are becoming increasingly ubiquitous [1,2,3,4]. Wireless sensor networks are being utilized in a diverse array of tracking and monitoring applications from environmental [5] and health monitoring [6] to traffic [7] and border surveillance [8]. In many of these applications, the nodes in the network are mobile and knowledge of their positions is a prerequisite for completing the task, and crucial for information sharing, data collection, and scheduling [9]. For example, if the locations of the nodes are unknown or significantly incorrect, the data they have collected from surrounding environment, such as wildlife [10] or weather information [11], will be useless, since the positional information is not available.

Localization algorithms estimate the locations of unknown nodes in the network using the positions of a known subset of the nodes to provide the required positional information. The most widely used localization techniques in the literature are distance-based localization algorithms, such as trilateration, radio interference positioning system (RIPS) [12], and the Hop-Distance algorithm [13]. These algorithms estimate the inter-node distances and require anchors, which is nodes with known locations, to provide the locations of the remaining nodes. The location of the anchor nodes is accessed via global positioning system (GPS) or a priori information [12,14,15,16,17,18].

While, GPS is widely used in locating unknown nodes in many situations, such as indoor, urban, and forest environments, the positions of nodes are difficult to obtain from GPS [19,20,21,22]. In this case, the anchors are absent or the positions of the anchors are not available, and the above algorithms cannot be applied. This is widely regarded as the most significant challenge in the positioning and navigation field [23,24,25]. Therefore, there is an increasing need for anchorless localization of sensor networks for use in GPS-denied or contested environments. Using the movement of nodes and the inter-node communication, cooperative localization can be leveraged in order to solve this problem. This scenario arises in the field of robotics swarms, especially in the navigation and formation control of unmanned aerial vehicles swarms under a GPS-denied environment [20,25,26,27].

In practice, cooperative localization can be achieved by utilizing the inter-node distances [28]. However, relative localization only gives node positions that satisfy the distance constraint, which means that there could be ambiguity problems, i.e., ambiguity due to rotation and/or flip [29]. The multidimensional scaling (MDS) algorithm is a widely used algorithm that is capable of tackling the anchorless localization problem [30,31,32,33,34,35]. The aim of MDS is to represent the similarity (or dissimilarity) of high dimensional data in a lower dimensional map which describes the relative distances between pairs of objects (in this case sensor nodes). Like other relative localization methods MDS can also be subject to the ambiguity problem; hence, algorithms have been proposed to attempt to address this problem. In [36] a MDS-based algorithm using moving nodes is presented, which constructs a cost function involving velocities and inter-node distances of all nodes at two consecutive time instants. In [37], a similar algorithm is proposed to solve the anchorless localization problem for nodes that can estimate the position via a nonlinear least square estimator; however, in this case, only one node is moving.

In this paper, we present an efficient algorithm for anchorless cooperative localization that is based on MDS. The algorithm mitigates the rotation and flip problems by taking advantage of the movement and inter-node communication of the mobile nodes. Unlike existing algorithms that operate in an iterative manner the proposed algorithm presents a closed-form solution that is computationally efficient. The algorithm is first derived in the noise-free case and the theoretical result is given. Subsrquently, the noisy case is considered and the associated closed-form estimator is presented. The proposed algorithm is supported by a rigorous theoretical derivation which provides optimal parameters. The simulation results support the theoretical analysis and indicate that the algorithm outperforms alternative methods.

This paper is organized, as follows: Section 2 introduces the background to the MDS algorithm and the associated ambiguity problem. The theoretical solution to the ambiguity problem is then presented in Section 3. Based on the theoretical analysis, Section 4 describes the impact of noise and introduces the proposed closed-form estimator for determining positions in noisy scenarios. Section 5 presents simulations for validating the proposed algorithm and, finally, Section 6 concludes the paper.

## 2. Multidimensional Scaling Algorithm

In this section, we briefly introduce the MDS algorithm and the ambiguity problem in an ideal scenario. As stated previously, the goal of MDS is to find a representation of the data that provides a low dimensional map (usually two or three dimensions) of the relative positions of the nodes based on their pairwise distances. If we consider the two-dimensional case where there are *n* nodes with their true coordinates denoted by $S_{i}={\left[x_{i},y_{i}\right]}^{T}$ where i=1,…,n and n≥3. Afterwards, in the noise-free case the distance between nodes *i* and *j*, where i,j=1,…,n and i≠j is given by $d_{i,j}$. If we assume in the ideal scenario that the nodes are able to measure the true distances between each other so that the pairwise distance between two nodes with coordinates $S_{i}$ and $S_{j}$ is given by

$$d_{i,j}=∥S_{i}-S_{j}∥=\sqrt{{\left(S_{i}-S_{j}\right)}^{T}\left(S_{i}-S_{j}\right)},$$

and, furthermore, the squared distance $d_{i,j}^{2}$ can be written as

$$d_{i,j}^{2}=S_{i}^{T}S_{i}-2S_{i}^{T}S_{j}+S_{j}^{T}S_{j},$$

then we have the following symmetric Euclidean distance matrix:

(1) $$D=\left[\begin{array}{ccccc} 0 & d_{1,2}^{2} & d_{1,3}^{2} & \cdots & d_{1,n}^{2}\\ d_{2,1}^{2} & 0 & d_{2,3}^{2} & \cdots & d_{2,n}^{2}\\ d_{3,1}^{2} & d_{3,2}^{2} & 0 & \cdots & d_{3,n}^{2}\\ \vdots & \vdots & \vdots & \ddots & \vdots \\ d_{n,1}^{2} & d_{n,2}^{2} & d_{n,3}^{2} & \cdots & 0 \end{array}\right].$$

If $S=[S_{1},\cdots ,S_{n}]$ is the collection of all of the node coordinates and ψ is the diagonal elements of $S^{T}S$, i.e.,

$$\psi =\mathrm{diag}\left(S^{T}S\right)={\left[s_{1}^{T}s_{1},\ldots ,s_{n}^{T}s_{n}\right]}^{T},$$

then we can rewrite D as

(2) $$D=\psi e^{T}-2S^{T}S+e\psi^{T},$$

where $e={\left[1,\ldots ,1\right]}^{T}$ is the vector of ones of length *n*. Using the centering operation $H=I-ee^{T}/n$, then we have

$$-\frac{1}{2}HDH=U\Lambda U^{T}.$$

where $U\Lambda U^{T}$ is the eigendecomposition of the symmetric matrix $-\frac{1}{2}HDH$. Then we can recover S (up to a translation and orthogonal transformation) via the following formula

(3) $$S^{*}=\Lambda^{\frac{1}{2}}U^{T},$$

MDS is an efficient algorithm for resolving the relative positions of the nodes [32]. However, as is apparent from the above analysis, in the absence of anchor nodes, MDS can only give relative positions of the nodes, which can include rotation and flip ambiguity. In other words, the result of MDS maintains the relative inter-node distances; however, these calculated locations of the nodes may be flipped and/or rotated versions of the true positions of the nodes. Obviously, when considering navigation of mobile nodes or formation control of the sensor network, incorrect positions of the nodes can lead to problems.

### The Ambiguity Problem

In order to consider the ambiguity problem, we assume a set of n nodes in 2D Euclidean space. We fix a coordinate system, which, without a loss of generality, places the first node $s_{1}$ at the origin: $s_{1}={\left[0,0\right]}^{T}$. We recall that knowledge of the distances provides an ambiguity up to a universal Euclidean transformation of the nodes. This fixing of node 1 at the origin removes the shift from this Euclidean transformation. Accordingly, the solution of the MDS, $S^{*}$, is replaced by subtracting $s_{1}^{*}$ from each column, so that, with some abuse of notation, $s_{1}^{*}={\left[0,0\right]}^{T}$, and the other $s_{j}^{*}$ become $s_{j}^{*}-s_{1}^{*}$. Once the shift is removed, the remaining ambiguity devolves to a rotation and a reflection (flip). We give the definitions of rotation and flip ambiguities, as follows.

**Definition** **1** (Rotation ambiguity). *If there exists an angle θ≠2kπ, k∈Z such that*

(4) $$S=M\left(\theta \right)S^{*},$$

*where M(θ) is rotation matrix with angle θ and it is defined by [38]*

(5) $$M\left(\theta \right)=\left[\begin{array}{cc} \cos(\theta ) & \sin(\theta )\\ -\sin(\theta ) & \cos(\theta ) \end{array}\right],$$

*then rotation ambiguity occurs.*

**Definition** **2** (Flip ambiguity). *Flip ambiguity occurs if*

(6) $$S=FS^{*}\;\;\;\mathrm{with}\;\;\;F=\left[\begin{array}{cc} -1 & 0\\ 0 & 1 \end{array}\right].$$

**Remark** **1.** *The matrix F can be defined equivalently by $F=\left[\begin{array}{cc} 1 & 0\\ 0 & -1 \end{array}\right].$ This definition can be obtained via simply rotating (6) by π. In the following analysis, we use the definition of F as in Definition 2.*

It can be seen that rotation ambiguity and flip ambiguity can occur simultaneously. If this is the case, then the true positions can be represented by

(7) $$S=M\left(\theta \right)FS^{*}.$$

**Figure 1:**
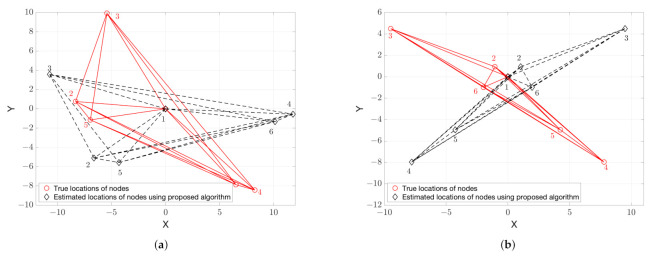
Illustration of the positions of nodes calculated via multidimensional scaling (MDS), where red circles are the desired position (randomly generated) and black diamonds are the output of MDS. (**a**) rotation ambiguity only; and, (**b**) rotation and flip ambiguities.

Figure 1 shows samples of these two ambiguities.

## 3. Resolving Rotation and Flip Ambiguities

In this section, the rotation ambiguity is analyzed mathematically in the noise-free scenario and an analytical solution to the rotation and flip ambiguities presented.

### 3.1. Analysis of Rotation Ambiguity

Firstly, we assume that there only exists rotation ambiguity between S and $S^{*}$, no flip ambiguity. This means that based on the coordination rotation principle [38], $S^{*}$ can be rotated to S using an unknown angle θ via (4). Hence, the true (unknown) locations of the nodes ${\left[x_{i},y_{i}\right]}^{T}$, for i=1,…,n, can be obtained by rotating ${\left[x_{i}^{*},y_{i}^{*}\right]}^{T}$ by θ, i.e.,

(8) $$\begin{array}{c} S=M\left(\theta \right)S^{*}. \end{array}$$

Therefore, we need to know θ to obtain the true locations of the nodes. In order to achieve this, we allow the lead node to move; this movement can then be utilized to obtain information that can be used to recover the locations of other nodes. Consider mobile nodes that are equipped with inertial navigation systems, the lead node can move with known displacement and orientation; that is, we let the lead node move to a known position, i.e., ${\left[\Delta x_{1}^{\prime },\Delta y_{1}^{\prime }\right]}^{T}$. Subsequently, the coordinates of all nodes after moving can be obtained as

(9) $$\begin{array}{cc} S^{\prime } & =S+\Delta S^{\prime } \end{array}$$

where the *i*-th column of $\Delta S^{\prime }$ is given by

(10) $$\Delta S_{i}^{\prime }=\left\{\begin{array}{cc} {\left[\Delta x_{1}^{\prime },\;\Delta y_{1}^{\prime }\right]}^{T} & i=1\\ {\left[0,\;0\right]}^{T} & i\neq 1 \end{array}\right.,$$

Accordingly, after moving the distance between the *i*th and *j*th nodes is

$$d_{i,j}^{\prime }=∥S_{i}^{\prime }-S_{j}^{\prime }∥,$$

where $S_{i}^{\prime }$ is the *i*th column of $S^{\prime }$. We can then update the distance matrix (2) with the entries $d_{i,j}^{{}^{\prime }\;2}$ to give

(11) $$\begin{array}{cc} D^{\prime } & =\psi^{\prime }e^{T}-2{S^{\prime }}^{T}S^{\prime }+e{\psi^{\prime }}^{T}, \end{array}$$

where, by considering $S_{1}={\left[0,0\right]}^{T}$ and $\Delta S_{i}^{\prime }={\left[0,0\right]}^{T}$ for i=2,…,n,

(12) $$\begin{array}{c} \psi^{\prime }=\mathrm{diag}\left(S^{\prime T}S^{\prime }\right)=\mathrm{diag}\left(S^{T}S\right)+\mathrm{diag}\left(\Delta {S^{\prime }}^{T}\Delta S^{\prime }\right)≜\psi +\Delta \psi^{\prime } \end{array}$$

Substituting (9) and (12) into the distance matrix (11) we have

$$\begin{array}{cc} D^{\prime } & =\left(\psi +\Delta \psi^{\prime }\right)e^{T}-2{\left(S+\Delta S^{\prime }\right)}^{T}\left(S+\Delta S^{\prime }\right)+e{\left(\psi +\Delta \psi^{\prime }\right)}^{T}\\ \; & =\left(\psi e^{T}-2S^{T}S+e\psi^{T}\right)+\left(\Delta \psi^{\prime }e^{T}-2\Delta {S^{\prime }}^{T}\Delta S^{\prime }+e\Delta {\psi^{\prime }}^{T}\right)-2\Delta {S^{\prime }}^{T}S-2S^{T}\Delta S^{\prime }\\ \; & =D+\Delta D^{\prime }-2\Delta {S^{\prime }}^{T}S-2S^{T}\Delta S^{\prime }, \end{array}$$

giving

(13) $$\begin{array}{cc} 0 & =D-D^{\prime }+\Delta D^{\prime }-2\Delta {S^{\prime }}^{T}S-2S^{T}\Delta S^{\prime }, \end{array}$$

where 0 is a zero matrix with dimensions n×n. This equation describes the relationship between both the locations and distance matrices pre and post the lead node moving; this information can be used to obtain the angle of rotation θ. If we break the analysis of (13) into three parts, then we have the following:

1. Since only the lead node’s position is changed, then the term $D-D^{\prime }$ in (13) becomes
  (14) $$D-D^{\prime }=\left[\begin{array}{cccc} 0 & d_{1,2}^{2}-d_{1,2}^{{}^{\prime }\;2} & \cdots & d_{1,n}^{2}-d_{1,n}^{{}^{\prime }\;2}\\ d_{1,2}^{2}-d_{1,2}^{{}^{\prime }\;2} & 0 & \cdots & 0\\ \vdots & \vdots & \ddots & \vdots \\ d_{1,n}^{2}-d_{1,n}^{{}^{\prime }\;2} & 0 & \cdots & 0 \end{array}\right].$$
2. In (13), ΔD is the distance matrix between point $\Delta S_{1}$ and n−1 origin points ${\left[0,0\right]}^{T}$, i.e.,
  (15) $$\Delta D^{\prime }=\left[\begin{array}{cccc} 0 & \Delta {x_{1}^{\prime }}^{2}+\Delta {y_{1}^{\prime }}^{2} & \cdots & \Delta {x_{1}^{\prime }}^{2}+\Delta {y_{1}^{\prime }}^{2}\\ \Delta {x_{1}^{\prime }}^{2}+\Delta {y_{1}^{\prime }}^{2} & 0 & \cdots & 0\\ \vdots & \vdots & \ddots & \vdots \\ \Delta {x_{1}^{\prime }}^{2}+\Delta {y_{1}^{\prime }}^{2} & 0 & \cdots & 0 \end{array}\right].$$
3. For the term $-2\Delta {S^{\prime }}^{T}S-2S^{T}\Delta S^{\prime }$ in (13), since $S^{T}\Delta S^{\prime }$ can be calculated by
  (16) $$S^{T}\Delta S^{\prime }=\left[\begin{array}{cc} 0 & 0\\ x_{2} & y_{2}\\ x_{3} & y_{3}\\ \vdots & \vdots \\ x_{n} & y_{n} \end{array}\right]\left[\begin{array}{ccccc} \Delta x_{1}^{\prime } & 0 & 0 & \cdots & 0\\ \Delta y_{1}^{\prime } & 0 & 0 & \cdots & 0 \end{array}\right]=\left[\begin{array}{cccc} 0 & 0 & \cdots & 0\\ x_{2}\Delta x_{1}^{\prime }+y_{2}\Delta y_{1}^{\prime } & 0 & \cdots & 0\\ \vdots & \vdots & \ddots & \vdots \\ x_{n}\Delta x_{1}^{\prime }+y_{n}\Delta y_{1}^{\prime } & 0 & \cdots & 0 \end{array}\right],$$
  therefore
  (17) $$-\left(2\Delta {S^{\prime }}^{T}S+2S^{T}\Delta S^{\prime }\right)=-2\left({\left(S^{T}\Delta S^{\prime }\right)}^{T}+S^{T}\Delta S^{\prime }\right).$$
  Inserting the rotation (8) into $x_{i}\Delta x_{1}^{\prime }+y_{i}\Delta y_{1}^{\prime }$ in (16), for i=2,…,n, we have
  (18) $$\begin{array}{cc} x_{i}\Delta x_{1}^{\prime }+y_{i}\Delta y_{1}^{\prime } & =x_{i}^{*}\Delta x_{1}^{\prime }\cos\left(\theta \right)+y_{i}^{*}\Delta x_{1}^{\prime }\sin\left(\theta \right)+y_{i}^{*}\Delta y_{1}^{\prime }\cos\left(\theta \right)-x_{i}^{*}\Delta y_{1}^{\prime }\sin\left(\theta \right)\\ \; & =\left(x_{i}^{*}\Delta x_{1}^{\prime }+y_{i}^{*}\Delta y_{1}^{\prime }\right)\cos\left(\theta \right)+\left(y_{i}^{*}\Delta x_{1}^{\prime }-x_{i}^{*}\Delta y_{1}^{\prime }\right)\sin\left(\theta \right). \end{array}$$
  Using (18) allows us to express (17) in a way that is independent of ${\left[x_{i},\;y_{i}\right]}^{T}$.

If we combine (14)–(18), then (13) becomes

(19) $$D-D^{\prime }+\Delta D^{\prime }-2\Delta {S^{\prime }}^{T}S-2S^{T}\Delta S^{\prime }=\left[\begin{array}{cccc} 0 & f_{2}\left(\theta \right) & \cdots & f_{n}\left(\theta \right)\\ f_{2}\left(\theta \right) & 0 & \cdots & 0\\ \vdots & \vdots & \ddots & \vdots \\ f_{n}\left(\theta \right) & 0 & \cdots & 0 \end{array}\right]=0,$$

where

(20) $$\begin{array}{c} f_{i}\left(\theta \right)=a_{i}+b_{i}\cos\left(\theta \right)+c_{i}\sin\left(\theta \right),\;\;i=2,\ldots ,n \end{array}$$

with coefficients

(21) $$\begin{array}{cc} a_{i} & =d_{1,i}^{2}-d_{1,i}^{{}^{\prime }\;2}+\Delta {x_{1}^{\prime }}^{2}+\Delta {y_{1}^{\prime }}^{2} \end{array}$$

(22) $$\begin{array}{cc} b_{i} & =-2(x_{i}^{*}\Delta x_{1}^{\prime }+y_{i}^{*}\Delta y_{1}^{\prime }) \end{array}$$

(23) $$\begin{array}{cc} c_{i} & =2(x_{i}^{*}\Delta y_{1}^{\prime }-y_{i}^{*}\Delta x_{1}^{\prime }) \end{array}$$

Equation (19) is equivalent to the following system of equations:

(24) $$\left\{\begin{array}{cc} a_{2}+b_{2}\cos\left(\theta \right)+c_{2}\sin\left(\theta \right)= & 0\\ \;\vdots & \vdots \\ a_{n}+b_{n}\cos\left(\theta \right)+c_{n}\sin\left(\theta \right)= & 0 \end{array}\right.,$$

Finally, the solution to (24) is the angle to resolve the rotation ambiguity. Importantly, this solution can be shown to be unique when n≥3. If we consider the case of n=3, then (24) can be expressed as

(25) $$\left\{\begin{array}{c} \sin\left(\theta \right)=\frac{a_{3}b_{2}-a_{2}b_{3}}{b_{3}c_{2}-b_{2}c_{3}}≜W_{1}\\ \cos\left(\theta \right)=\frac{a_{2}c_{3}-a_{3}c_{2}}{b_{3}c_{2}-b_{2}c_{3}}≜W_{2} \end{array}\right..$$

Obviously, given $W_{1}$ and $W_{2}$, (25) has a unique solution to θ within $\left[-\pi ,\pi \right)$. Similarly, it is straightforward to show that, when n≥3, (24) has a unique solution within $\left[-\pi ,\pi \right)$.

### 3.2. Analysis of Rotation and Flip Ambiguities

Having obtained a unique solution to the rotation angle when only rotation ambiguity is present, in this section we present an analytical solution that is based on the analysis in Section 3.1 for when rotation and flip ambiguities occur simultaneously. The key idea behind this method is again to use the mobility of the lead node to acquire extra information in order to detect the flip of the initial MDS localization result.

Firstly, we assume that there exist three non-collinear nodes in order to be able to detect flip ambiguity. Next, we note that the *i*th equation in (24) has the following solutions $\theta_{i,1}$ and $\theta_{i,2}$:

(26) $$\theta_{i,1,2}=atan2\left(a_{i}b_{i}\pm |c_{i}|\sqrt{b_{i}^{2}+c_{i}^{2}-a_{i}^{2}},\;a_{i}c_{i}\mp \frac{b_{i}}{c_{i}}\left|c_{i}\right|\sqrt{b_{i}^{2}+c_{i}^{2}-a_{i}^{2}}\right),$$

where atan2(·,·)∈[−π,π) is the 2-argument arctangent. Therefore, the solution to (26), which is common to all values of *i*, ∀i=2,…,n is the unique solution to (24). It can also be shown that $\theta_{i,1,2}\in \left[-\pi ,\pi \right)$ given in (26) can be rewritten and rearranged into a concise form

(27) $$\begin{array}{cc} \theta_{i,1} & =g\left(atan2\left(y_{i}^{*},x_{i}^{*}\right)-atan2\left(y_{i},x_{i}\right)\right) \end{array}$$

(28) $$\begin{array}{cc} \theta_{i,2} & =g\left(\theta_{i,1}+2\Theta_{i}\right) \end{array}$$

where $\Theta_{i}=atan2\left(y_{i},x_{i}\right)+atan2\left(\Delta x_{1}^{\prime },\Delta y_{1}^{\prime }\right)-\frac{\pi }{2}$ and the function $g\left(t\right)=t-2\pi \left\lfloor \frac{t}{2\pi }+\frac{1}{2}\right\rfloor$ can wrap any arbitrary angle *t* in radians into range [−π,π). Appendix A provides the full derivation of these equations.

**Figure 2:**
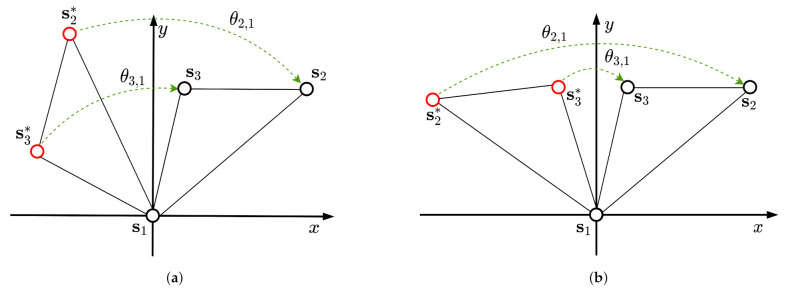
Illustration of angle $\theta_{i,1}$ given in (27) using three nodes with true locations $s_{i}$ and solved ambiguous locations $s_{i}^{*}$. (**a**) rotation ambiguity and $\theta_{2,1}=\theta_{3,1}$; (**b**) flip ambiguity and $\theta_{2,1}\neq \theta_{3,1}$.

The angles $\theta_{i,1}$ in (27) represent the angles between vectors $\vec{S_{1}S_{i}^{*}}$ and $\vec{S_{1}S_{i}}$ for i=2,…,n, as shown in Figure 2, and play an important role in the ability to detect flip ambiguity. When there is only rotation ambiguity, because of the uniqueness of solution of (24), we have $\theta_{i,1}=\theta_{j,1}$, ∀i,j=2,…,n, as shown in the previous section. Whereas, the angles $\theta_{i,2}$ in (28) are the summation of $\theta_{i,1}$ and the angle induced by $\Delta x_{1}^{\prime }$ and $\Delta x_{2}^{\prime }$. Obviously, if there exist three non-collinear nodes, $\Theta_{i}\neq \Theta_{j}$, ∀i,j=2,…,n and i≠j and, therefore, from (28) we have $\theta_{i,2}\neq \theta_{j,2}$, ∀i,j=2,…,n. In contrast, it can be shown that flip ambiguity exists if and only if $\theta_{i,1}\neq \theta_{j,1}$, ∀i,j=2,…,n and i≠j. In order to illustrate why this is the case, we give the counter example, assuming without loss of generality, that n=3, $\theta_{2,1}=\theta_{3,1}$ and flip ambiguity exists. $\theta_{2,1}=\theta_{3,1}$ implies that $S_{i}^{*}$, i=2,3, can be rotated simultaneously to the true positions $S_{i}$ via either $\theta_{2,1}$ or $\theta_{3,1}$, as shown in Figure 2a. Hence, this contradicts the assumption of the existence of flip ambiguity.

Furthermore, extending to the case where n>3, still assuming that $\theta_{2,1}=\theta_{3,1}$ and flip ambiguity exists. We know that, for i=2,3, $S_{i}^{*}$ can be simultaneously rotated to the true positions $S_{i}$ via $\theta_{2,1}$ (or $\theta_{3,1}$). Because there exist three non-collinear nodes, then three nodes with correct positions are sufficient for guaranteeing the localization of the whole network [39]. In this case, $s_{1}$, $s_{2}$ and $s_{3}$ can be found exactly from $\theta_{2,1}$ (or $\theta_{3,1}$). Therefore, $S_{i}$, for i=4,…,n, must be solvable via rotating $S_{i}^{*}$ by angle $\theta_{2,1}$ (or $\theta_{3,1}$) and, as a result, $\theta_{i,1}=\theta_{2,1}=\theta_{3,1}$. This again contradicts the assumption of existence of flip ambiguity. On the other hand, if $\theta_{i,1}\neq \theta_{j,1}$, ∀i,j=2,⋯,n and i≠j, then it is obvious that there exists flip ambiguity, since $s_{i}^{*}$ cannot be rotated to $S_{i}$ simultaneously. As a conclusion, there exists flip ambiguity if and only if $\theta_{i,1}\neq \theta_{j,1}$, ∀i,j=2,⋯,n and i≠j.

In what follows, we assume that there exist three non-collinear nodes and, based on the above analysis, we make the following conclusion:

(29) $$\forall i,j=2,\ldots ,n\;\mathrm{and}\;i\neq j\left\{\begin{array}{cc} \theta_{i,1}=\theta_{j,1}\left(\mathrm{equivalently}\;\theta_{i,2}\neq \theta_{j,2}\right), & \;\mathrm{If}\;\mathrm{no}\;\mathrm{flip}\;\mathrm{ambiguity}\;\mathrm{exists}.\\ \theta_{i,1}\neq \theta_{j,1}\left(\mathrm{equivalently}\;\theta_{i,2}=\theta_{j,2}\right), & \;\mathrm{If}\;\mathrm{flip}\;\mathrm{ambiguity}\;\mathrm{exists}. \end{array}\right.$$

Although we cannot use (27)–(29) directly to determine the existence of flip as they contain unknown true positions, those results are crucial in deriving the estimator for locations in noisy scenario.

In reality, the solution to (24) is computed using $S^{*}$, $\Delta S^{\prime }$ and $D^{\prime }$. We denote this unique solution by $\theta_{R}\left(S^{*},\Delta S^{\prime },D^{\prime }\right)$. The same notation $\theta_{R}\left(\cdot ,\cdot ,\cdot \right)$ is used to denote a rotation angle that is calculated with different variables, nonetheless, whatever the variables used, the method is the same as described above. If we denote the positions that are calculated using the rotation angle $\theta_{R}\left(S^{*},\Delta S^{\prime },D^{\prime }\right)$ as $S^{**}$ we have the following result

(30) $$S^{**}=M\left(\theta_{R}\left(S^{*},\Delta S^{\prime },D^{\prime }\right)\right)S^{*}=\left[\begin{array}{ccc} x_{1}^{**}, & x_{2}^{**}, & \cdots ,x_{n}^{**}\\ y_{1}^{**}, & y_{2}^{**}, & \cdots ,y_{n}^{**} \end{array}\right].$$

If the lead node then moves to a second position $\left[\Delta x_{1}^{\prime \prime },\Delta y_{1}^{\prime \prime }\right]$ giving a matrix formed by true positions $S^{\prime \prime }=S+\Delta S^{\prime \prime }$, where

(31) $$\Delta S^{\prime \prime }=\left[\Delta S_{1}^{\prime \prime },\Delta S_{2}^{\prime \prime },\ldots ,\Delta S_{n}^{\prime \prime }\right]=\left[\begin{array}{cccc} \Delta x_{1}^{\prime \prime } & 0 & \cdots & 0\\ \Delta y_{1}^{\prime \prime } & 0 & \cdots & 0 \end{array}\right].$$

After obtaining a new distance matrix $D^{\prime \prime }$ at position $S^{\prime \prime }$, we can solve $\theta_{R}\left(S^{**},\Delta S^{\prime \prime },D^{\prime \prime }\right)$. If $\theta_{R}\left(S^{**},\Delta S^{\prime \prime },D^{\prime \prime }\right)=0$, then there is no flip ambiguity and the true positions are $S=S^{**}$; otherwise, we move to the process of resolving the flip ambiguity. For this, according to Definition 2, all the values in $S^{*}$ along the x−axis are required to be flipped to obtain $FS^{*}$. Subsequently, we only need to calculate the rotation angle using $\theta_{R}\left(FS^{*},\Delta S^{\prime },D^{\prime }\right)$. It should be noted that, since $\Delta S^{\prime }$ and $D^{\prime }$ are fixed, it is unnecessary to take any new measurements and the true position S can be resolved by

(32) $$S=M\left(\theta_{R}\left(FS^{*},\Delta S^{\prime },D^{\prime }\right)\right)FS^{*}.$$

## 4. Proposed Algorithm Robust to Ambiguity and Noise

The analysis in the previous section assumes ideal measurements; however, in practice, the measurements are corrupted by noise that can have a significant impact on the localization performance. If we consider the distances between two nodes, in the noise-free case the distance between the *i*th and *j*th nodes is the same regardless of which node it is measured from, i.e., $d_{i,j}=d_{j,i}$. When noise is introduced, this is no longer the case, if we denote the measured distances as ${\bar{d}}_{i,j}$ and ${\bar{d}}_{j,i}$, then ${\bar{d}}_{i,j}\neq {\bar{d}}_{j,i}$ resulting in uncertainty in our estimates of the distances. In general, the measured distance between the *i*th and *j*th nodes can be modeled by

(33) $${\bar{d}}_{i,j}=d_{i,j}+\omega_{i,j},\;i,j=1,\ldots ,n,\;i\neq j,$$

where $d_{i,j}$ is the true distance and $\omega_{i,j}$ is the measurement noise.

Accordingly, the noisy Euclidean distance matrix (EDM) can be written into

(34) $$\bar{D}=\left[\begin{array}{ccccc} 0 & {\bar{d}}_{1,2}^{2} & {\bar{d}}_{1,3}^{2} & \cdots & {\bar{d}}_{1,n}^{2}\\ {\bar{d}}_{2,1}^{2} & 0 & {\bar{d}}_{2,3}^{2} & \cdots & {\bar{d}}_{2,n}^{2}\\ {\bar{d}}_{3,1}^{2} & {\bar{d}}_{3,2}^{2} & 0 & \cdots & {\bar{d}}_{3,n}^{2}\\ \vdots & \vdots & \vdots & \ddots & \vdots \\ {\bar{d}}_{n,1}^{2} & {\bar{d}}_{n,2}^{2} & {\bar{d}}_{n,3}^{2} & \cdots & 0 \end{array}\right].$$

As we know that $d_{i,j}=d_{j,i}$ when the noise is absent, therefore, in order to obtain a symmetric EDM in noisy case, we can use

(35) $${\hat{d}}_{i,j}={\hat{d}}_{j,i}=E\left({\bar{d}}_{i,j},{\bar{d}}_{j,i}\right)$$

where $E({\bar{d}}_{i,j},{\bar{d}}_{j,i})$ is an estimator for the distance between *i*-th and *j*-th nodes using ${\bar{d}}_{i,j}$ and ${\bar{d}}_{j,i}$. In practice, this estimator is designed by using the knowledge of noise distribution. For example, when the noise in ${\bar{d}}_{i,j}$ and ${\bar{d}}_{j,i}$ are assumed to comprise independent and identically distributed (i.i.d.) normal distributions, then we have ${\hat{d}}_{i,j}={\hat{d}}_{j,i}=\frac{1}{2}\left({\bar{d}}_{i,j}+{\bar{d}}_{j,i}\right)$ [40]. Because the study of the estimator $E({\bar{d}}_{i,j},{\bar{d}}_{j,i})$ and estimation of the EDM is outside the scope of this article, we refer the interested reader to [40,41] for more information, including completing and estimating an EDM.

Accordingly, the estimated symmetric EDM $\hat{D}$ can be obtained while using ${\hat{d}}_{i,j}$. Similarly, we can obtain ${\hat{D}}^{\prime }$ by using the new position of $S^{\prime }=S+\Delta S^{\prime }$, as described in (10).

In (24), in the noise-free case, the solution is unique and easy to find. However, in the noisy environment, the theoretical unique solution to (24) is not guaranteed. Therefore, a key issue in estimating θ is to find a value that satisfies a certain objective, i.e.,

(36) $${\hat{\theta }}_{R}\left(S^{*},\Delta S^{\prime },D^{\prime }\right)=\arg\min_{\theta \in [-\pi ,\pi )}Obj\left(\theta ;\;S^{*},\Delta S^{\prime },{\hat{D}}^{\prime }\right),$$

where $Obj\left(\theta ;\;S^{*},\Delta S^{\prime },D^{\prime }\right)$ is an objective function of θ given $S^{*}$, $\Delta S^{\prime }$, and ${\hat{D}}^{\prime }$. In general, the least square estimator provides a good choice of objective function, as it is a well defined computationally efficient estimator. The objective function based on the least square estimator is given by

(37) $$Obj\left(\theta ;\;S^{*},\Delta S^{\prime },D^{\prime }\right)=\sum_{i=1}^{n}{\left(a_{i}+b_{i}\cos\left(\theta \right)+c_{i}\sin\left(\theta \right)\right)}^{2}.$$

Solutions to (37) can be obtained by taking the derivative of the objective function with respect to θ and equating to zero, from this we obtain a quartic equation (see Appendix B for details), which has, at most, four real roots giving the corresponding collection of angles as

$$\theta_{\lambda }=\left\{\arcsin\left(\lambda_{1}\right),g\left(\pi -\arcsin\left(\lambda_{1}\right)\right),\ldots ,\arcsin\left(\lambda_{m}\right),g\left(\pi -\arcsin\left(\lambda_{m}\right)\right)\right\}\in \left[-\pi ,\pi \right),$$

where $\lambda_{j}\in \left[-1,1\right]$, j=1,…,m, and *m* is the number of solutions, such that 1≤m≤4. Subsequently, (37) becomes

(38) $${\hat{\theta }}_{R}\left(S^{*},\Delta S^{\prime },{\hat{D}}^{\prime }\right)=\arg\min_{\theta \in \theta_{\lambda }}Obj\left(\theta ;\;S^{*},\Delta S^{\prime },{\hat{D}}^{\prime }\right),$$

and, accordingly, by (30)

(39) $$S^{**}=M\left({\hat{\theta }}_{R}\left(S^{*},\Delta S^{\prime },{\hat{D}}^{\prime }\right)\right)S^{*}.$$

In order to detect flip ambiguity, we can follow the same method, as described in Section 3, i.e., let the lead node move to another position ${\left[\Delta x_{1}^{\prime \prime },\Delta y_{1}^{\prime \prime }\right]}^{T}$ and solve ${\hat{\theta }}_{R}(S^{**},$$\Delta S^{\prime \prime },{\hat{D}}^{\prime \prime })$ via (38) while using the estimated distance matrix ${\hat{D}}^{\prime \prime }$ obtained at the new position $S^{\prime \prime }=S+\Delta S^{\prime \prime }$. However, becasue of the presence of noise ${\hat{\theta }}_{R}\left(S^{**},\Delta S^{\prime \prime },{\hat{D}}^{\prime \prime }\right)$ is not necessarily equal to 0 when there is no flip ambiguity. To handle this, we need to create a detector for the flip. For this, we use a straightforward threshold detector:

(40) $$\left\{\begin{array}{cc} \mathrm{no}\;\mathrm{flip}, & \;\mathrm{if}\;\left|{\hat{\theta }}_{R}\left(S^{**},\Delta S^{\prime \prime },{\hat{D}}^{\prime \prime }\right)\right|\leq \left|l\right|\\ \mathrm{flip}, & \;\mathrm{if}\;\left|{\hat{\theta }}_{R}\left(S^{**},\Delta S^{\prime \prime },{\hat{D}}^{\prime \prime }\right)\right|>\left|l\right|, \end{array}\right.$$

The following proposition, the proof of which is given in Appendix C, allows for us to efficiently find an optimal threshold in (40).

**Proposition** **1.** *Under the noise-free case, if there exists flip ambiguity, then*

(41) $$\theta_{R}\left(S^{**},\Delta S^{\prime \prime },D^{\prime \prime }\right)=g\left(-2atan2\left(\Delta x_{1}^{\prime },\Delta y_{1}^{\prime }\right)+2atan2\left(\Delta x_{1}^{\prime \prime },\Delta y_{1}^{\prime \prime }\right)\right),$$

*where $g\left(t\right)=t-2\pi \left\lfloor \frac{t}{2\pi }+\frac{1}{2}\right\rfloor$.*

From Proposition 1, under the noise-free case, we know the value of $\theta_{R}\left(S^{**},\Delta S^{\prime \prime },D^{\prime \prime }\right)$ when a flip occurs; therefore, in the noisy case, the optimal threshold is

$$l=\frac{1}{2}g\left(-2atan2\left(\Delta x_{1}^{\prime },\Delta y_{1}^{\prime }\right)+2atan2\left(\Delta x_{1}^{\prime \prime },\Delta y_{1}^{\prime \prime }\right)\right).$$

As a conclusion, the positions of nodes can estimated using the following formula:

(42) $$\hat{S}=\left\{\begin{array}{cc} M\left({\hat{\theta }}_{R}\left(S^{*},\Delta S^{\prime },{\hat{D}}^{\prime }\right)\right)S^{*}, & \;\mathrm{if}\;\left|{\hat{\theta }}_{R}\left(S^{**},\Delta S^{\prime \prime },{\hat{D}}^{\prime \prime }\right)\right|\leq \left|l\right|\\ M\left({\hat{\theta }}_{R}\left(FS^{*},\Delta S^{\prime },{\hat{D}}^{\prime }\right)\right)FS^{*}, & \;\mathrm{if}\;\left|{\hat{\theta }}_{R}\left(S^{**},\Delta S^{\prime \prime },{\hat{D}}^{\prime \prime }\right)\right|>\left|l\right| \end{array}\right.$$

Algorithm 1 summarizes the algorithm to estimate locations of nodes with noisy measurements.
**Algorithm 1:** Algorithm to estimate locations of mobile nodes.
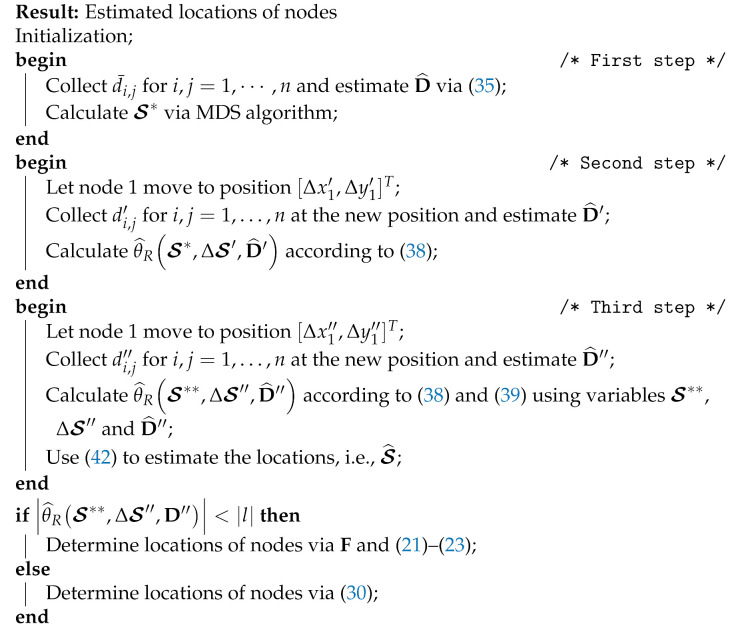


## 5. Simulations

Networks with randomly generated nodes were used to test the performance of the proposed algorithm. In all of the following simulations the position of the first node is fixed to [0,0] and the positions of the other nodes are uniformly generated within [−20,20].

**Figure 3:**
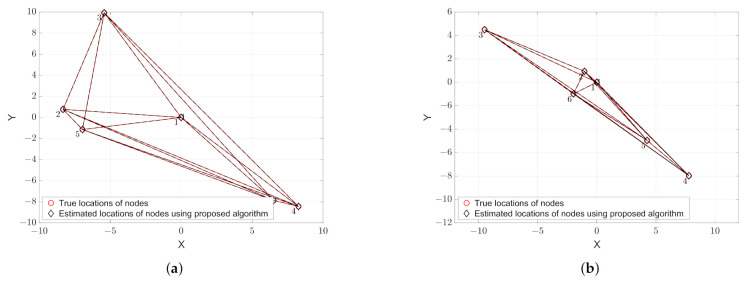
Estimation of the positions of nodes in noise-free scenarios. (**a**) Rotation only; and (**b**) Rotation and flip.

To validate the proposed approach that can address the ambiguity problem, we use the same noise-free scenarios as given in Figure 1 the results of which are shown in Figure 3. Unlike the results that are obtained from the MDS alone, as shown in Figure 1, when we compare the locations of the true and estimated nodes in Figure 3, we can see that, while using the method that is presented in Section 3, the rotation and flip ambiguities are solved correctly and, therefore, the positions of the nodes are successfully recovered.

**Figure 4:**
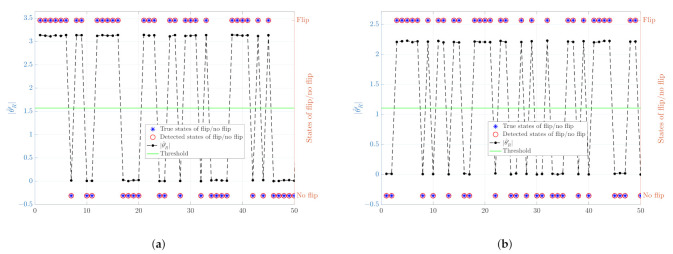
An illustration of the detection of flip/no flip states and the corresponding values of $\left|{\hat{\theta }}_{R}\left(S^{**},\Delta S^{\prime \prime },{\hat{D}}^{\prime \prime }\right)\right|$. The left y-axes represent the values of $\left|{\hat{\theta }}_{R}\left(S^{**},\Delta S^{\prime \prime },{\hat{D}}^{\prime \prime }\right)\right|$ and the threshold while the right y-axes show the true and detected states of flip/no flip with σ=0.01. (**a**) The number of nodes n=6, $\left[\Delta x_{1}^{\prime },\Delta y_{1}^{\prime }\right]=\left[1,0\right]$ and $\left[\Delta x_{1}^{\prime \prime },\Delta y_{1}^{\prime \prime }\right]=\left[0,1\right]$; (**b**) The number of nodes n=10, $\left[\Delta x_{1}^{\prime },\Delta y_{1}^{\prime }\right]=\left[1,0.5\right]$ and $\left[\Delta x_{1}^{\prime \prime },\Delta y_{1}^{\prime \prime }\right]=\left[0,1\right]$.

Next, we consider the noisy scenario, as discussed in Section 4, in the noisy scenario the detection of whether flip has occurred is no longer straightforward and requires an appropriate choice of threshold. Firstly, to demonstrate the ability of the proposed algorithm to detect flip or no flip states, we tested two different network configurations: one with the number of nodes n=6 and the other with n=10. The noise is assumed to be Gaussian distributed with mean 0 and standard deviation, σ, of 0.01. For each of the configurations, the outcomes of 50 simulations are shown in Figure 4. The results show that the proposed algorithm can correctly detect flip ambiguity in the noisy scenario.

Having established that the proposed algorithm can successfully deal with the ambiguity problem, we now consider the performance of the algorithm in terms of the accuracy of the localization. For the sake of simplicity, we define the noise level, standard variance of the noise, as $\delta =-20\log_{10}\sigma$, with δ chosen to range from 20 to 50 in steps of five and σ calculated accordingly. For each noise level, the Root-Mean-Square Error (RMSE) from 2000 simulations is used to evaluate the performance of the localization. The RMSE is defined by

(43) $$\begin{array}{c} \mathrm{RMSE}=\frac{1}{n}\sum_{i=1}^{n}\sqrt{\sum_{j=1}^{m}\frac{{\left({\hat{x}}_{i,j}-x_{i,j}\right)}^{2}+{\left({\hat{y}}_{i,j}-y_{i,j}\right)}^{2}}{m}} \end{array}$$

where $\left(x_{i,j},y_{i,j}\right)$ and $\left({\hat{x}}_{i,j},{\hat{y}}_{i,j}\right)$ are the true and estimated location of *i*-th, i=1,⋯,n, node in *j*-th, j=1,⋯,m, Monte Carlo simulation. It is assumed that $\left(x_{i,j},y_{i,j}\right)$ is generated within region [−100,100]×[−100,100] uniformly in each Monte Carlo simulation.

In order to provide a comparison with the proposed algorithm, we implemented the nonlinear least squares estimator (NLSE) that is presented in [37], solving the nonlinear least square problem using an optimization method. Additionally, the proposed algorithm essentially takes advantage of the mobility of the node to create virtual anchors for localizing the unknown nodes. As a result, the alignment method of relative locations proposed in [30,42] can be potentially applied in this scenario. As a comparison, the performance of this conventional method is also given.

**Figure 5:**
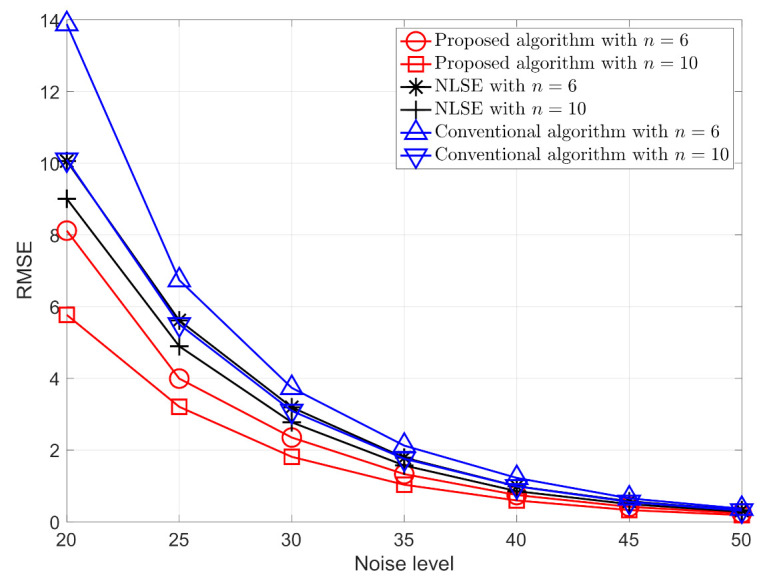
The Root-Mean-Square Error (RMSE) for the proposed algorithm, the NLS estimator [37], NLSE, and conventional algorithm proposed in [30,42] for different numbers of nodes and different noise levels.

From the simulation results that are shown in Figure 5, it can be seen that the proposed algorithm has better performance in terms of the RMSE for different noise levels and numbers of nodes than the NLS estimator with the conventional method. The localization error are arise from two effects: 1. MDS localization error; 2. mis-alignment error, i.e., the error from inaccurately aligning the positions. Additionally, it should also be noted that the algorithm proposed in this paper can simultaneously estimate the positions of all of the nodes, which cannot be achieved using the algorithm that was presented in [37]. As an indication of the computational efficiency of the proposed algorithm, our simulations indicate a ratio of required CPU time of the proposed algorithm relative to NLSE is Proposed algorithm:NLSE = 1:8.83.

## 6. Conclusions

In this paper, we have presented an efficient cooperative localization algorithm that is based on MDS. The algorithm provides a practical solution for anchorless localization of mobile nodes using noisy measurements. Unlike traditional MDS algorithms, which suffer from an ambiguity problem, the algorithm that is presented here can solve the flip and rotation ambiguities and accurately estimate the positions of nodes in 2D space. The simulation results demonstrate the accuracy of the algorithm, showing that it outperforms alternative methods. At the same time, the proposed algorithm provides greater efficiency than alternative solutions that operate in an iterative manner by providing a closed-form solution. We point out that the main limitations of this algorithm are twofold. Firstly, as mentioned above, this algorithm has been developed in a 2D scenario, which limits its application in more complex situations. Although one may follow a similar procedure to derive the corresponding algorithm for a more general case, i.e., 3D space, this is non-trivial, as the geometry of the network has more degrees of freedom in the 3D space. Secondly, and in a similar vein to other algorithms, the algorithm proposed here requires inertial navigation to provide the displacement of the moving node. Consequently, a deeper analysis of the error that arises from the inertial navigation system should be taken into account in improving this algorithm. These issues will be addressed in future work.

## Data Availability

Not applicable.

## Abbreviations

The following abbreviations are used in this manuscript:

$d_{i,j}$	The distance between nodes *i* and *j*
D	Euclidean distance matrix given the collection of locations S
M(θ)	Rotation matrix for given angle θ
$S_{i}={\left[x_{i},y_{i}\right]}^{T}$	True coordinates of the *i*th node
$S=[S_{1},\cdots ,S_{n}]$	Collection of the true locations of all of the nodes
$S^{*}$	Output of multidimensional scaling given the distance matrix D
$S^{**}$	Rotation of the output of multidimensional scaling, $S^{*}$, using rotation matrix M(θ) with the angle, θ, obtained from the function $\theta_{R}\left(S^{*},\Delta S^{\prime },D^{\prime }\right)$
$\theta_{R}\left(S^{*},\Delta S^{\prime },D^{\prime }\right)$	Function to solve the possible rotation angle between the true positions and $S^{*}$ using the associated parameters
ψ	The diagonal elements of $S^{T}S$

The following notation is used to denote changes in the variables:

${\left(\cdot \right)}^{\prime }$	The parameter after the first movement
${\left(\cdot \right)}^{\prime \prime }$	The parameter after the second movement
$\Delta {\left(\cdot \right)}^{\prime }$	The change in the parameter after the first movement
$\Delta {\left(\cdot \right)}^{\prime \prime }$	The change in the parameter, relative to the original, after the second movement

For example, $S^{\prime }$ is the collection of true locations of all of the nodes incorporating the changes in position, i.e., $S^{\prime }=S+\Delta S^{\prime }$, similarly $S^{\prime \prime }=S+\Delta S^{\prime \prime }$. It should be noted that $S^{\prime \prime }=S+\Delta S^{\prime \prime }$ can be re-formulated as $S^{\prime \prime }=S^{\prime }+\bar{\Delta S^{\prime \prime }}$ with $\bar{\Delta S^{\prime \prime }}≜\Delta S^{\prime \prime }-\Delta S^{\prime }$.

## Appendix A

**Proposition** **A1.**
*Function $g\left(t\right)=t-2\pi \left\lfloor \frac{t}{2\pi }+\frac{1}{2}\right\rfloor$ can wrap arbitrary angle t in radians into range [−π,π) and it satisfies following properties: P.1 $g\left(t_{1}\pm t_{2}\right)=g\left(g\left(t_{1}\right)\pm g\left(t_{2}\right)\right)$; and P.2 g(−t)=−g(t).*

**Lemma** **A1.**
*Let $g\left(t\right)=t-2\pi \left\lfloor \frac{t}{2\pi }+\frac{1}{2}\right\rfloor$, then*

(A1) $$atan2\left(\beta_{1}\alpha_{2}\pm \beta_{2}\alpha_{1},\;\alpha_{1}\alpha_{2}\mp \beta_{1}\beta_{2}\right)=g\left(atan2\left(\beta_{1},\alpha_{1}\right)\pm atan2\left(\beta_{2},\alpha_{2}\right)\right),$$

From (21)–(23), we have

$$\begin{array}{cc} a_{i} & =d_{1,i}^{2}-d_{1,i}^{\prime \;2}+\Delta {x_{1}^{\prime }}^{2}+\Delta {y_{1}^{\prime }}^{2}\\ \; & =\left(x_{i}^{2}+y_{i}^{2}\right)-\left({\left(x_{i}-\Delta x_{1}^{\prime }\right)}^{2}+{\left(y_{i}-\Delta y_{1}^{\prime }\right)}^{2}\right)+\Delta {x_{1}^{\prime }}^{2}+\Delta {y_{1}^{\prime }}^{2}\\ \; & =2\left(x_{i}\Delta x_{1}^{\prime }+y_{i}\Delta y_{1}^{\prime }\right) \end{array}$$

and

(A2) $$\begin{array}{cc} b_{i}^{2}+c_{i}^{2}-a_{i}^{2}= & 4{\left(x_{i}^{*}\Delta x_{1}^{\prime }+y_{i}^{*}\Delta y_{1}^{\prime }\right)}^{2}+4{\left(y_{i}^{*}\Delta x_{1}^{\prime }-x_{i}^{*}\Delta y_{1}^{\prime }\right)}^{2}-4{\left(x_{i}\Delta x_{1}^{\prime }+y_{i}\Delta y_{1}^{\prime }\right)}^{2}\\ = & 4\left(\left(d_{1,i}^{2}-d_{1,i}^{2}+y_{i}^{2}\right)\Delta {x_{1}^{\prime }}^{2}+\left(d_{1,i}^{2}-d_{1,i}^{2}+x_{i}^{2}\right)\Delta {y_{1}^{\prime }}^{2}-2x_{i}y_{i}\Delta x_{1}^{\prime }\Delta y_{1}^{\prime }\right)\\ = & 4{\left(x_{i}\Delta y_{1}^{\prime }-y_{i}\Delta x_{1}^{\prime }\right)}^{2}. \end{array}$$

Then from Lemma A1 and (A2), we have

$$\begin{array}{cc} \theta_{i,1,2} & =atan2\left(-a_{i}c_{i}\mp \frac{b_{i}}{c_{i}}|c_{i}|\sqrt{b_{i}^{2}+c_{i}^{2}-a_{i}^{2}},\;-a_{i}b_{i}\pm \left|c_{i}\right|\sqrt{b_{i}^{2}+c_{i}^{2}-a_{i}^{2}}\right) \end{array}$$

(A3) $$\begin{array}{cc} \; & =-g\left(atan2\left(b_{i},c_{i}\right)+atan2\left(a_{i},\;\pm \frac{|c_{i}|}{c_{i}}\sqrt{b_{i}^{2}+c_{i}^{2}-a_{i}^{2}}\right)\right) \end{array}$$

(A4) $$\begin{array}{cc} \; & =-g\left(atan2\left(b_{i},c_{i}\right)+atan2\left(a_{i},\;\pm 2\left(x_{i}\Delta y_{1}-y_{i}\Delta x_{1}\right)\right)\right). \end{array}$$

From (A3) to (A4), the sign of $\pm \frac{|c_{i}|}{c_{i}}\sqrt{4{\left(x_{i}\Delta y_{1}-y_{i}\Delta x_{1}\right)}^{2}}$ is interpreted by ±, which implies that the values of $\theta_{i,1}$ and $\theta_{i,2}$ may be simply exchanged.

Furthermore,

(A5) $$\begin{array}{cc} atan2(b_{i},c_{i}) & =atan2\left(-\left(x_{i}^{*}\Delta x_{1}^{\prime }+y_{i}^{*}\Delta y_{1}^{\prime }\right),\;x_{i}^{*}\Delta y_{1}^{\prime }-y_{i}^{*}\Delta x_{1}^{\prime }\right)\\ \; & =-atan2\left(x_{i}^{*}\Delta x_{1}^{\prime }+y_{i}^{*}\Delta y_{1}^{\prime },\;x_{i}^{*}\Delta y_{1}^{\prime }-y_{i}^{*}\Delta x_{1}^{\prime }\right)\\ \; & =g\left(-atan2\left(y_{i}^{*},x_{i}^{*}\right)-atan2\left(\Delta x_{1}^{\prime },\Delta y_{1}^{\prime }\right)\right) \end{array}$$

and

(A6) $$\begin{array}{cc} atan2\left(a_{i},\;\pm 2\left(x_{i}\Delta y_{1}^{\prime }-y_{i}\Delta x_{1}^{\prime }\right)\right) & =g\left(atan2\left(2\left(x_{i}\Delta x_{1}^{\prime }+y_{i}\Delta y_{1}^{\prime }\right),\;\pm 2\left(x_{i}\Delta y_{1}^{\prime }-y_{i}\Delta x_{1}^{\prime }\right)\right)\right)\\ \; & =\left\{\begin{array}{c} g\left(atan2\left(y_{i},x_{i}\right)+atan2\left(\Delta x_{1}^{\prime },\Delta y_{1}^{\prime }\right)\right)\\ g\left(-atan2\left(y_{i},x_{i}\right)-atan2\left(\Delta x_{1}^{\prime },\Delta y_{1}^{\prime }\right)\right) \end{array}\right.. \end{array}$$

Substituting (A6) and (A5) into (A4) leads to

$$\begin{array}{cc} \theta_{i,1} & =g\left(atan2\left(y_{i}^{*},x_{i}^{*}\right)-atan2\left(y_{i},x_{i}\right)\right)\\ \theta_{i,2} & =g\left(atan2\left(y_{i}^{*},x_{i}^{*}\right)+atan2\left(y_{i},x_{i}\right)+2atan2\left(\Delta x_{1}^{\prime },\Delta y_{1}^{\prime }\right)-\pi \right)\\ \; & =g\left(\theta_{i,1}+2\Theta_{i}\right) \end{array}$$

where $\Theta_{i}=atan2\left(y_{i},x_{i}\right)+atan2\left(\Delta x_{1}^{\prime },\Delta y_{1}^{\prime }\right)-\frac{\pi }{2}$.

## Appendix B

Taking the derivative of $Obj\left(\theta ;\;S^{*},\Delta S^{\prime },D^{\prime }\right)$ with respect to θ, we have

(A7) $$\begin{array}{cc} \; & \frac{\partial }{\partial \theta }\sum_{i=1}^{n}{\left(a_{i}+b_{i}\cos\left(\theta \right)+c_{i}\sin\left(\theta \right)\right)}^{2}\\ = & \sum_{i=1}^{n}2\left(c_{i}\cos\left(\theta \right)-b_{i}\sin\left(\theta \right)\right)\left(a_{i}+b_{i}\cos\left(\theta \right)+c_{i}\sin\left(\theta \right)\right)\\ = & \sum_{i=1}^{n}\left(-a_{i}b_{i}\sin\left(\theta \right)+a_{i}c_{i}\cos\left(\theta \right)+b_{i}c_{i}\cos^{2}\left(\theta \right)-b_{i}c_{i}\sin^{2}\left(\theta \right)+\left(c_{i}^{2}-b^{2}\right)\sin\left(\theta \right)\cos\left(\theta \right)\right)\\ = & 2\left(-\alpha_{n}\sin\left(\theta \right)+\beta_{n}\cos\left(\theta \right)+2\gamma_{n}\cos^{2}\left(\theta \right)+\delta_{n}\sin\left(\theta \right)\cos\left(\theta \right)-\gamma_{n}\right), \end{array}$$

where

$$\begin{array}{cccc} \alpha_{n} & =\sum_{i=1}^{n}a_{i}b_{i}, & \beta_{n} & =\sum_{i=1}^{n}a_{i}c_{i},\\ \gamma_{n} & =\sum_{i=1}^{n}b_{i}c_{i}, & \delta_{n} & =\sum_{i=1}^{n}\left(c_{i}^{2}-b_{i}^{2}\right). \end{array}$$

If we let sin(θ)=λ∈[−1,1] and $\cos\left(\theta \right)=\pm \sqrt{1-\lambda^{2}}$. Then setting (A7) to 0 leads to:

(A8) $$\gamma_{n}\pm \beta_{n}\sqrt{1-\lambda^{2}}-2\gamma_{n}\lambda^{2}\pm \delta_{n}\lambda \sqrt{1-\lambda^{2}}-\alpha_{n}\lambda =0.$$

Rearranging (A8) and squaring both sides, we have:

(A9) $$\begin{array}{cc} \; & {\left(\pm \left(\beta_{n}+\delta_{n}\lambda \right)\sqrt{1-\lambda^{2}}\right)}^{2}={\left(-\gamma_{n}+2\gamma_{n}\lambda^{2}+\alpha_{n}\lambda \right)}^{2}\\ ⟹\; & A_{n}\lambda^{4}+B_{n}\lambda^{3}+C_{n}\lambda^{2}+D_{n}\lambda +E_{n}=0, \end{array}$$

where

$$\begin{array}{cccccc} A_{n} & =4\gamma_{n}^{2}+\delta_{n}^{2}, & B_{n} & =2(2\alpha_{n}\gamma_{n}+\beta_{n}\delta_{n}), & C_{n} & =\alpha_{n}^{2}+\beta_{n}^{2}-4\gamma_{n}^{2}-\delta_{n}^{2},\\ D_{n} & =2(-\alpha_{n}\gamma_{n}-\beta_{n}\delta_{n}), & E_{n} & =-\beta_{n}^{2}+\gamma_{n}^{2}. \end{array}$$

Equation (A9) is a quartic equation [43] and has at most 4 real roots. Suppose that we have 1≤m≤4 solution(s) from (A9) satisfying $\lambda_{j}\in \left[-1,1\right]$, j=1,…,m, then the corresponding collection of angles is

$$\theta_{\lambda }=\left\{\arcsin\left(\lambda_{1}\right),g\left(\pi -\arcsin\left(\lambda_{1}\right)\right),\ldots ,\arcsin\left(\lambda_{m}\right),g\left(\pi -\arcsin\left(\lambda_{m}\right)\right)\right\}\in \left[-\pi ,\pi \right).$$

## Appendix C

**Proof** **of Proposition 1.** If we recall from (29) that in the case of flip ambiguity we have

(A10) $$\theta_{R}\left(S^{*},\Delta S^{\prime },D^{\prime }\right)=g\left(atan2\left(y_{i}^{*},x_{i}^{*}\right)+atan2\left(y_{i},x_{i}\right)+2atan2\left(\Delta x_{1}^{\prime },\Delta y_{1}^{\prime }\right)-\pi \right),$$

then we can rearrange to give

(A11) $$\begin{array}{cc} \theta_{R}\left(S^{*},\Delta S^{\prime },D^{\prime }\right)= & g(atan2\left(y_{i}^{*},x_{i}^{*}\right)+atan2\left(y_{i},x_{i}\right)+2atan2\left(\Delta x_{1}^{\prime },\Delta y_{1}^{\prime }\right)-\pi \\ \; & +atan2\left(y_{i}^{**},x_{i}^{**}\right)-atan2\left(y_{i}^{**},x_{i}^{**}\right))\\ = & g\left(atan2\left(y_{i}^{**},x_{i}^{**}\right)+atan2\left(y_{i},x_{i}\right)+2atan2\left(\Delta x_{1}^{\prime },\Delta y_{1}^{\prime }\right)-\pi \right)\\ \; & +g\left(-atan2\left(y_{i}^{**},x_{i}^{**}\right)+atan2\left(y_{i}^{*},x_{i}^{*}\right)\right). \end{array}$$

From the definition of $S^{**}$ in (30) it can be shown that

$$g\left(-atan2\left(y_{i}^{**},x_{i}^{**}\right)+atan2\left(y_{i}^{*},x_{i}^{*}\right)\right)=\theta_{R}\left(S^{*},\Delta S^{\prime },D^{\prime }\right),$$

giving

(A12) $$\begin{array}{cc} \theta_{R}\left(S^{*},\Delta S^{\prime },D^{\prime }\right)= & g\left(atan2\left(y_{i}^{**},x_{i}^{**}\right)+atan2\left(y_{i},x_{i}\right)+2atan2\left(\Delta x_{1}^{\prime },\Delta y_{1}^{\prime }\right)-\pi \right)+\\ \; & \theta_{R}\left(S^{*},\Delta S^{\prime },D^{\prime }\right)\\ 0= & g\left(atan2\left(y_{i}^{**},x_{i}^{**}\right)+atan2\left(y_{i},x_{i}\right)+2atan2\left(\Delta x_{1}^{\prime },\Delta y_{1}^{\prime }\right)-\pi \right). \end{array}$$

Incorporating (A12) into the equation for $\theta_{R}\left(S^{**},\Delta S^{\prime \prime },D^{\prime \prime }\right)$ we have

(A13) $$\begin{array}{cc} \theta_{R}\left(S^{**},\Delta S^{\prime \prime },D^{\prime \prime }\right)= & g\left(atan2\left(y_{i}^{**},x_{i}^{**}\right)+atan2\left(y_{i},x_{i}\right)+2atan2\left(\Delta x_{1}^{\prime \prime },\Delta y_{1}^{\prime \prime }\right)-\pi \right)\\ = & g(atan2\left(y_{i}^{**},x_{i}^{**}\right)+atan2\left(y_{i},x_{i}\right)+2atan2\left(\Delta x_{1}^{\prime \prime },\Delta y_{1}^{\prime \prime }\right)-\pi \\ \; & +2atan2\left(\Delta x_{1}^{\prime },\Delta y_{1}^{\prime }\right)-2atan2\left(\Delta x_{1}^{\prime },\Delta y_{1}^{\prime }\right))\\ = & g\left(atan2\left(y_{i}^{**},x_{i}^{**}\right)+atan2\left(y_{i},x_{i}\right)+2atan2\left(\Delta x_{1}^{\prime },\Delta y_{1}^{\prime }\right)-\pi \right)\\ \; & +g\left(-2atan2\left(\Delta x_{1}^{\prime },\Delta y_{1}^{\prime }\right)+2atan2\left(\Delta x_{1}^{\prime \prime },\Delta y_{1}^{\prime \prime }\right)\right)\\ = & g\left(0\right)+g\left(-2atan2\left(\Delta x_{1}^{\prime },\Delta y_{1}^{\prime }\right)+2atan2\left(\Delta x_{1}^{\prime \prime },\Delta y_{1}^{\prime \prime }\right)\right)\\ = & g\left(-2atan2\left(\Delta x_{1}^{\prime },\Delta y_{1}^{\prime }\right)+2atan2\left(\Delta x_{1}^{\prime \prime },\Delta y_{1}^{\prime \prime }\right)\right). \end{array}$$

□

## References

[1] Ramson S.R.J., Moni D.J. Applications of Wireless Sensor Networks—A Survey. Proceedings of the International Conference on Innovations in Electrical, Electronics, Instrumentation and Media Technology (ICEEIMT).

[2] Yick J., Mukherjee B., Ghosal D. (2008). Wireless Sensor Network Survey. Comput. Netw..

[3] Mao G., Fidan B., Anderson B.D. (2007). Wireless Sensor Network Localization Techniques. Comput. Netw..

[4] Chong C.Y., Kumar S. (2003). Sensor Networks: Evolution, Opportunities, and Challenges. Proc. IEEE.

[5] Lombardo L., Corbellini S., Parvis M., Elsayed A., Angelini E., Grassini S. (2018). Wireless Sensor Network for Distributed Environmental Monitoring. IEEE Trans. Instrum. Meas..

[6] Alemdar H., Ersoy C. (2010). Wireless Sensor Networks for Healthcare: A Survey. Comput. Netw..

[7] Kafi M.A., Challal Y., Djenouri D., Doudou M., Bouabdallah A., Badache N. (2013). A Study of Wireless Sensor Networks for Urban Traffic Monitoring: Applications and Architectures. Procedia Comput. Sci..

[8] Hammoudeh M., Al-Fayez F., Lloyd H., Newman R., Adebisi B., Bounceur A., Abuarqoub A. (2017). A Wireless Sensor Network Border Monitoring System: Deployment Issues and Routing Protocols. IEEE Sens. J..

[9] Moran B., Suvorova S., Howard S., Byrnes J., Ostheimer G. (2006). Sensor Management for Radar: A Tutorial. Advances in Sensing with Security Applications.

[10] Garcia-Sanchez A.J., Garcia-Sanchez F., Losilla F., Kulakowski P., Garcia-Haro J., Rodríguez A., López-Bao J.V., Palomares F. (2010). Wireless Sensor Network Deployment for Monitoring Wildlife Passages. Sensors.

[11] Abdullah S., Bertalan S., Masar S., Coskun A., Kale I. A Wireless Sensor Network for Early Forest Fire Detection and Monitoring as a Decision Factor in the Context of a Complex Integrated Emergency Response System. Proceedings of the IEEE Workshop on Environmental, Energy, and Structural Monitoring Systems (EESMS).

[12] Maróti M., Völgyesi P., Dóra S., Kusý B., Nádas A., Lédeczi Á., Balogh G., Molnár K. (2005). Radio Interferometric Geolocation. Proceedings of the 3rd International Conference on Embedded Networked Sensor Systems (SenSys).

[13] Nath S., Ekambaram V.N., Kumar A., Kumar P.V. (2012). Theory and Algorithms for Hop-Count-Based Localization with Random Geometric Graph Models of Dense Sensor Networks. ACM Trans. Sens. Netw..

[14] Wang X., Moran B., Brazil M. Hyperbolic Positioning Using RIPS Measurements for Wireless Sensor Networks. Proceedings of the 15th IEEE International Conference on Networks.

[15] Wymeersch H., Lien J., Win M.Z. (2009). Cooperative Localization in Wireless Networks. Proc. IEEE.

[16] Yaghoubi F., Abbasfar A.A., Maham B. (2014). Energy-Efficient RSSI-Based Localization for Wireless Sensor Networks. IEEE Commun. Lett..

[17] Tomic S., Beko M., Dinis R. (2015). RSS-Based Localization in Wireless Sensor Networks Using Convex Relaxation: Noncooperative and Cooperative Schemes. IEEE Trans. Veh. Technol..

[18] Goel S., Kealy A., Lohani B. (2016). Cooperative UAS Localization Using Lowcost Sensors. ISPRS Ann. Photogramm. Remote Sens. Spatial Inf. Sci..

[19] Ahrens S., Levine D., Andrews G., How J.P. Vision-Based Guidance and Control of a Hovering Vehicle in Unknown, GPS-Denied Environments. Proceedings of the IEEE International Conference on Robotics and Automation.

[20] Bachrach A., Prentice S., He R., Roy N. (2011). RANGE-Robust autonomous navigation in GPS-denied environments. J. Field Robot..

[21] Zhang L., Ye M., Anderson B.D., Sarunic P., Hmam H. Cooperative localisation of UAVs in a GPS-denied environment using bearing measurements. Proceedings of the IEEE 55th Conference on Decision and Control (CDC).

[22] Balamurugan G., Valarmathi J., Naidu V.P.S. Survey on UAV navigation in GPS denied environments. Proceedings of the 2016 International Conference on Signal Processing, Communication, Power and Embedded System (SCOPES).

[23] de Paula Veronese L., Cheein F.A., Bastos-Filho T., Souza A.F.D., de Aguiar E. (2015). A Computational Geometry Approach for Localization and Tracking in GPS-denied Environments. J. Field Robot..

[24] Schnipke E., Reidling S., Meiring J., Jeffers W., Hashemi M., Tan R., Nemati A., Kumar M. Autonomous Navigation of UAV through GPS-Denied Indoor Environment with Obstacles. Proceedings of the AIAA Infotech @ Aerospace.

[25] Russell J.S., Ye M., Anderson B.D., Hmam H., Sarunic P. (2017). Cooperative Localisation of a GPS-Denied UAV in 3-Dimensional Space Using Direction of Arrival Measurements. IFAC PapersOnLine.

[26] Singh S., Sujit P. (2016). Landmarks based path planning for UAVs in GPS-denied areas. IFAC PapersOnLine.

[27] Power W., Pavlovski M., Saranovic D., Stojkovic I., Obradovic Z. (2020). Autonomous Navigation for Drone Swarms in GPS-Denied Environments Using Structured Learning. Artificial Intelligence Applications and Innovations.

[28] Cao M., Anderson B.D.O., Morse A.S. (2006). Sensor Network Localization with Imprecise Distances. Syst. Control Lett..

[29] Beck B., Baxley R. Anchor Free Node Tracking Using Ranges, Odometry, and Multidimensional Scaling. Proceedings of the IEEE International Conference on Acoustics, Speech and Signal Processing (ICASSP).

[30] Ji X., Zha H. Sensor Positioning in Wireless Ad-Hoc Sensor Networks Using Multidimensional Scaling. Proceedings of the IEEE INFOCOM 2004.

[31] Borg I., Groenen P.J.F. (2005). Modern Multidimensional Scaling.

[32] Costa J.A., Patwari N., Hero A.O. (2006). Distributed Weighted-Multidimensional Scaling for Node Localization in Sensor Networks. ACM Trans. Sens. Netw..

[33] Rajan R.T., van der Veen A.J. (2015). Joint Ranging and Synchronization for an Anchorless Network of Mobile Nodes. IEEE Trans. Signal Process..

[34] Wei M., Aragues R., Sagues C., Calafiore G.C. (2015). Noisy Range Network Localization Based on Distributed Multidimensional Scaling. IEEE Sens. J..

[35] Saeed N., Nam H., Al-Naffouri T.Y., Alouini M. (2019). A State-of-the-Art Survey on Multidimensional Scaling-Based Localization Techniques. IEEE Commun. Surv. Tutor..

[36] Di Franco C., Melani A., Marinoni M. Solving Ambiguities in MDS Relative Localization. Proceedings of the International Conference on Advanced Robotics (ICAR).

[37] Guo K., Qiu Z., Meng W., Xie L., Teo R. (2017). Ultra-wideband based cooperative relative localization algorithm and experiments for multiple unmanned aerial vehicles in GPS denied environments. Int. J. Micro Air Veh..

[38] Arfken G.B., Weber H.J., Harris F.E. (2013). Mathematical Methods for Physicists.

[39] Anderson B.D.O., Shames I., Mao G., Fidan B. (2010). Formal Theory of Noisy Sensor Network Localization. SIAM J. Discret. Math..

[40] Zhang H., Liu Y., Lei H. (2019). Localization From Incomplete Euclidean Distance Matrix: Performance Analysis for the SVD–MDS Approach. IEEE Trans. Signal Process..

[41] Dokmanic I., Parhizkar R., Ranieri J., Vetterli M. (2015). Euclidean Distance Matrices: Essential theory, algorithms, and applications. IEEE Signal Process. Mag..

[42] Di Franco C., Bini E., Marinoni M., Buttazzo G.C. Multidimensional Scaling Localization with Anchors. Proceedings of the IEEE International Conference on Autonomous Robot Systems and Competitions (ICARSC).

[43] Strobach P. (2010). The Fast Quartic Solver. J. Comput. Appl. Math..

